# Supplementary material for: Smooth Interpolating Curves with Local Control and Monotone Alternating Curvature
Source: Comput Graph Forum. 2022 Oct 6;41(5):25–38. doi: 10.1111/cgf.14600 (PMC9827861; doi:10.1111/cgf.14600)
Supplement: Supplementary file 1 — Supplement Material [file CGF-41-25-s001.zip › Local-Smooth-Interpolating-MonoCurvature/extern/clothoids/submodules/quarticRootsFlocke/docs/api-cpp/function_a00023_1ac18c1444f6f1642ac85d6021bf837952.html]

Function PolynomialRoots::roots — Quartic Roots v1.0.0

### Navigation

- index
- toc
- next
- previous
- Quartic Roots »
- C++ API »
- Function PolynomialRoots::roots

# Function PolynomialRoots::roots¶

- Defined in File PolynomialRoots-Jenkins-Traub.cc

## Function Documentation¶

int PolynomialRoots::roots(valueType const \*op, indexType Degree, valueType \*zeror, valueType \*zeroi)¶
:   Find roots of a generic polynomial using Jenkins-Traub method

    Parameters
    :   - **op** – **[in]** the coefficients of the polynomial
        - **Degree** – **[in]** degree of the polynomial
        - **zeror** – **[out]** real part of the roots
        - **zeroi** – **[out]** imaginary part of the roots

    Returns
    :   error code, 0 OK

### Quick search

### Table of Contents

- Quartic Roots Solver
- C++ API

«
hide menu

menu
sidebar
»

### Navigation

- index
- toc
- next
- previous
- Quartic Roots »
- C++ API »
- Function PolynomialRoots::roots

© Copyright 2021, Enrico Bertolazzi.
Created using Sphinx 4.2.0.
